# Diazido­bis­[4,4,5,5-tetra­methyl-2-(1,3-thia­zol-2-yl)-2-imidazoline-1-oxyl 3-oxide-κ^2^ *N* ^1^,*O* ^3^]nickel(II)

**DOI:** 10.1107/S1600536810042455

**Published:** 2010-10-30

**Authors:** Jiu Li Chang, Zhi Yong Gao, Ning Zhao

**Affiliations:** a College of Chemistry and Environmental Science, Henan Normal University, Xinxiang 453002, People’s Republic of China; b School of Chemistry and Chemical Engineering, Henan Institute of Science and Technology, Xinxiang 453003, People’s Republic of China

## Abstract

In the title compound, [Ni(N_3_)_2_(C_10_H_14_N_3_O_2_S)_2_], the Ni^II^ atom lies on an inversion center and adopts a distorted *trans*-NiO_2_N_4_ octa­hedral geometry, coordinated by two *N*,*O*-bidentate 4,4,5,5-tetra­methyl-2-(5-methyl­imidazol-4-yl)-2-imidazoline-1-oxyl 3-oxide nitronyl nitroxide radical ligands and two monodentate azide anions.

## Related literature

For general background to mol­ecular magnetic materials and metal-radical magnetic materials, see: Vostrikova *et al.* (2000 ▶); Fegy *et al.* (1998 ▶); Kahn *et al.* (2000 ▶); Omata *et al.* (2001 ▶); Yamamoto *et al.* (2001 ▶); Fursova *et al.* (2003 ▶); Sroh *et al.* (2003 ▶); Chang *et al.* (2009 ▶); Schatzschneider *et al.* (2001 ▶). For the synthesis of nitronyl nitroxide radical ligands and the title compound, see: Ullman *et al.* (1970 ▶, 1972 ▶).
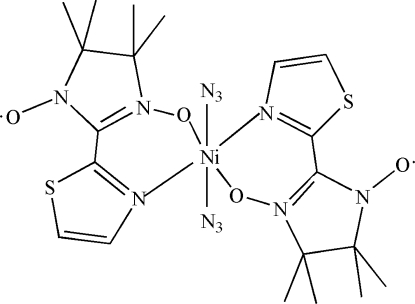


## Experimental

### 

#### Crystal data

[Ni(N_3_)_2_(C_10_H_14_N_3_O_2_S)_2_]
*M* *_r_* = 623.37
Monoclinic, 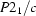

*a* = 9.9212 (7) Å
*b* = 12.1732 (8) Å
*c* = 11.1795 (8) Å
β = 102.695 (1)°
*V* = 1317.17 (16) Å^3^
*Z* = 2
Mo *K*α radiation
μ = 0.95 mm^−1^
*T* = 291 K
0.40 × 0.22 × 0.15 mm

#### Data collection

Bruker SMART APEX CCD diffractometer
Absorption correction: multi-scan (*SADABS*; Sheldrick, 1996 ▶) *T*_min_ = 0.703, *T*_max_ = 0.874
7812 measured reflections
3005 independent reflections
2834 reflections with *I* > 2σ(*I*)
*R*_int_ = 0.010

#### Refinement

*R*[*F* ^2^ > 2σ(*F* ^2^)] = 0.024
*wR*(*F* ^2^) = 0.065
*S* = 1.06
3005 reflections
182 parameters
H-atom parameters constrained
Δρ_max_ = 0.25 e Å^−3^
Δρ_min_ = −0.27 e Å^−3^

### 

Data collection: *SMART* (Bruker, 2002 ▶); cell refinement: *SAINT* (Bruker, 2002 ▶); data reduction: *SAINT*; program(s) used to solve structure: *SHELXS97* (Sheldrick, 2008 ▶); program(s) used to refine structure: *SHELXL97* (Sheldrick, 2008 ▶); molecular graphics: *SHELXTL* (Sheldrick, 2008 ▶); software used to prepare material for publication: *publCIF* (Westrip, 2010 ▶).

## Supplementary Material

Crystal structure: contains datablocks I, global. DOI: 10.1107/S1600536810042455/sj5040sup1.cif

Structure factors: contains datablocks I. DOI: 10.1107/S1600536810042455/sj5040Isup2.hkl

Additional supplementary materials:  crystallographic information; 3D view; checkCIF report

## Figures and Tables

**Fig. 1.**
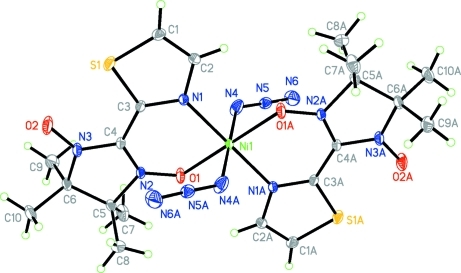
ORTEP drawing of the title compound with atom labeling. The thermal ellipsoids are drawn at 30% probability level [symmetry codes relating the atoms with and without the suffix A: -x + 1, -y + 2, -z + 1]

## supplementary crystallographic information

### Comment

The synthesis and study of transition metal complexes incorporating organic free radicals is a major research focus in the field of molecular magnetism (Vostrikova *et al.*, 2000; Fegy *et al.*, 1998; Kahn *et al.*, 2000; Omata *et al.*, 2001). In this field, nitronyl nitroxides acting as useful paramagnetic building blocks have been extensively used to assemble molecular magnetic materials, because many of them are good stable spin carriers even when coordinated to metal ions (Yamamoto *et al.*, 2001; Fursova *et al.*, 2003; Sroh *et al.*, 2003; Chang *et al.*, 2009; Schatzschneider *et al.*, 2001). We report herein the synthesis and crystal structure of one such nickel complex.

The asymmetric unit of the title compound (Fig. 1) contains half molecule. The Ni^II^ atom, lying on a an inversion center, is six-coordinated in a distorted octahedral geometry by two N atoms and two O atoms from two 4,4,5,5-tetramethyl-2-(5-methylimidazol-4-yl)-2- imidazoline-1-oxyl-3-oxide nitronyl nitroxide radical ligands and two N atoms from two azide anions.

### Experimental

The nitronyl nitroxide radical(2-(2'-thiazole)-4,4,5,5-tetramethylimidazoline-1-oxyl-3- oxide)was synthesized according to literature procedures (Ullman *et al.* 1970; Ullman *et al.* 1972). A mixed solution of nitronyl nitroxide radical ligands (2.00 mmol) and Ni(Ac)_2_.4H_2_O (1 mmol) in ethanol (10 ml) was added to an aqueous solution(10 mL) of NaN_3_(2 mmol) and the resulting mixed solution was stirred for one hour at room temperature and then filtered off. This filtrate was left to evaporate slowly. After one week, deep purple crystals suitable for X-ray analysis were isolated.

### Refinement

All C—H atoms were positioned geometrically, with C—H = 0.93 or 0.96 Å and constrained to ride on their parent atoms with *U*_iso_(H)=1.2U (carrier) or *U*_iso_ (H)=1.5U (methyl carrier).

### Crystal data

**Table:**

[Ni(N_3_)_2_(C_10_H_14_N_3_O_2_S)_2_]	*F*(000) = 648
*M**_r_* = 623.37	*D*_x_ = 1.572 Mg m^−^^3^
Monoclinic, *P*2_1_/*c*	Mo *K*α radiation, λ = 0.71073 Å
Hall symbol: -P 2ybc	Cell parameters from 5712 reflections
*a* = 9.9212 (7) Å	θ = 2.7–29.3°
*b* = 12.1732 (8) Å	µ = 0.95 mm^−^^1^
*c* = 11.1795 (8) Å	*T* = 291 K
β = 102.695 (1)°	Block, dark purple
*V* = 1317.17 (16) Å^3^	0.40 × 0.22 × 0.15 mm
*Z* = 2	

### Data collection

**Table:**

Bruker SMART APEX CCD diffractometer	3005 independent reflections
Radiation source: fine-focus sealed tube	2834 reflections with *I* > 2σ(*I*)
graphite	*R*_int_ = 0.010
φ and ω scans	θ_max_ = 27.5°, θ_min_ = 2.7°
Absorption correction: multi-scan (*SADABS*; Sheldrick , 1996)	*h* = −12→9
*T*_min_ = 0.703, *T*_max_ = 0.874	*k* = −14→15
7812 measured reflections	*l* = −14→13

### Refinement

**Table:**

Refinement on *F*^2^	Primary atom site location: structure-invariant direct methods
Least-squares matrix: full	Secondary atom site location: difference Fourier map
*R*[*F*^2^ > 2σ(*F*^2^)] = 0.024	Hydrogen site location: inferred from neighbouring sites
*wR*(*F*^2^) = 0.065	H-atom parameters constrained
*S* = 1.06	*w* = 1/[σ^2^(*F*_o_^2^) + (0.031*P*)^2^ + 0.5195*P*] where *P* = (*F*_o_^2^ + 2*F*_c_^2^)/3
3005 reflections	(Δ/σ)_max_ = 0.001
182 parameters	Δρ_max_ = 0.25 e Å^−^^3^
0 restraints	Δρ_min_ = −0.27 e Å^−^^3^

### Special details

**Table:**

Geometry. All e.s.d.'s (except the e.s.d. in the dihedral angle between two l.s. planes)are estimated using the full covariance matrix. The cell e.s.d.'s are takeninto account individually in the estimation of e.s.d.'s in distances, anglesand torsion angles; correlations between e.s.d.'s in cell parameters are onlyused when they are defined by crystal symmetry. An approximate (isotropic)treatment of cell e.s.d.'s is used for estimating e.s.d.'s involving l.s. planes.
Refinement. Refinement of *F*^2^ against ALL reflections. The weighted *R*-factor *wR* and goodness of fit *S* are based on *F*^2^, conventional *R*-factors *R* are based on *F*, with *F* set to zero for negative *F*^2^. The threshold expression of *F*^2^ > σ(*F*^2^) is used only for calculating *R*-factors(gt) *etc*. and is not relevant to the choice of reflections for refinement. *R*-factors based on *F*^2^ are statistically about twice as large as those based on *F*, and *R*- factors based on ALL data will be even larger.

### Fractional atomic coordinates and isotropic or equivalent isotropic displacement parameters (Å^2^)

**Table:**

	*x*	*y*	*z*	*U*_iso_*/*U*_eq_	
Ni1	0.5000	1.0000	0.5000	0.02603 (8)	
S1	0.75033 (4)	1.27825 (3)	0.38125 (4)	0.04133 (11)	
O1	0.64775 (12)	0.90740 (9)	0.43831 (12)	0.0486 (3)	
O2	0.89398 (15)	1.14747 (11)	0.24875 (14)	0.0611 (4)	
N1	0.60808 (11)	1.13921 (9)	0.47330 (10)	0.0272 (2)	
N2	0.72751 (11)	0.94577 (9)	0.37164 (10)	0.0285 (2)	
N3	0.84655 (12)	1.05745 (10)	0.28101 (11)	0.0360 (3)	
N4	0.61887 (17)	0.99070 (14)	0.67735 (14)	0.0568 (4)	
N5	0.58855 (14)	0.95757 (10)	0.76655 (12)	0.0380 (3)	
N6	0.5618 (2)	0.92594 (14)	0.85593 (15)	0.0652 (5)	
C1	0.64979 (16)	1.32436 (12)	0.47626 (15)	0.0391 (3)	
H1	0.6423	1.3976	0.4976	0.047*	
C2	0.58239 (15)	1.24038 (11)	0.51644 (13)	0.0329 (3)	
H2A	0.5231	1.2506	0.5694	0.039*	
C3	0.69761 (13)	1.14629 (10)	0.40176 (12)	0.0269 (2)	
C4	0.75220 (13)	1.05162 (11)	0.35158 (12)	0.0273 (3)	
C5	0.79699 (14)	0.86855 (11)	0.29890 (12)	0.0312 (3)	
C6	0.90436 (14)	0.94677 (12)	0.25934 (13)	0.0337 (3)	
C7	0.8572 (2)	0.77211 (15)	0.37997 (18)	0.0535 (5)	
H7A	0.9144	0.7992	0.4548	0.080*	
H7B	0.9117	0.7275	0.3378	0.080*	
H7C	0.7834	0.7288	0.3984	0.080*	
C8	0.68251 (19)	0.82790 (16)	0.19319 (16)	0.0524 (4)	
H8A	0.6110	0.7937	0.2256	0.079*	
H8B	0.7201	0.7755	0.1452	0.079*	
H8C	0.6447	0.8890	0.1424	0.079*	
C9	1.04886 (16)	0.94066 (17)	0.34192 (17)	0.0527 (4)	
H9A	1.1050	0.9986	0.3207	0.079*	
H9B	1.0898	0.8709	0.3310	0.079*	
H9C	1.0427	0.9486	0.4260	0.079*	
C10	0.9140 (2)	0.93844 (17)	0.12542 (15)	0.0534 (4)	
H10A	0.8244	0.9506	0.0736	0.080*	
H10B	0.9463	0.8666	0.1099	0.080*	
H10C	0.9772	0.9929	0.1084	0.080*	

### Atomic displacement parameters (Å^2^)

**Table:**

	*U*^11^	*U*^22^	*U*^33^	*U*^12^	*U*^13^	*U*^23^
Ni1	0.02752 (13)	0.02314 (12)	0.03225 (13)	−0.00064 (8)	0.01700 (9)	0.00075 (8)
S1	0.0393 (2)	0.02794 (18)	0.0629 (2)	−0.00619 (14)	0.02443 (18)	0.00477 (16)
O1	0.0554 (7)	0.0281 (5)	0.0793 (8)	0.0016 (5)	0.0519 (6)	0.0040 (5)
O2	0.0673 (8)	0.0448 (7)	0.0892 (10)	−0.0049 (6)	0.0564 (8)	0.0112 (6)
N1	0.0276 (5)	0.0250 (5)	0.0319 (5)	0.0002 (4)	0.0125 (4)	0.0003 (4)
N2	0.0263 (5)	0.0278 (5)	0.0360 (6)	0.0008 (4)	0.0166 (4)	−0.0009 (4)
N3	0.0350 (6)	0.0362 (6)	0.0439 (6)	−0.0003 (5)	0.0238 (5)	0.0031 (5)
N4	0.0533 (9)	0.0743 (11)	0.0408 (8)	−0.0261 (8)	0.0058 (7)	0.0125 (7)
N5	0.0436 (7)	0.0299 (6)	0.0405 (7)	−0.0034 (5)	0.0091 (5)	0.0021 (5)
N6	0.1006 (14)	0.0528 (9)	0.0498 (9)	−0.0085 (9)	0.0332 (9)	0.0077 (7)
C1	0.0374 (7)	0.0253 (6)	0.0563 (9)	−0.0016 (5)	0.0136 (7)	−0.0045 (6)
C2	0.0341 (7)	0.0276 (6)	0.0392 (7)	0.0018 (5)	0.0128 (6)	−0.0042 (5)
C3	0.0244 (6)	0.0252 (6)	0.0326 (6)	−0.0015 (5)	0.0098 (5)	0.0023 (5)
C4	0.0242 (6)	0.0302 (6)	0.0300 (6)	−0.0003 (5)	0.0114 (5)	0.0021 (5)
C5	0.0307 (6)	0.0327 (7)	0.0337 (6)	0.0056 (5)	0.0145 (5)	−0.0028 (5)
C6	0.0293 (7)	0.0421 (8)	0.0339 (7)	0.0037 (6)	0.0159 (5)	−0.0027 (6)
C7	0.0550 (10)	0.0485 (10)	0.0647 (11)	0.0239 (8)	0.0300 (9)	0.0160 (8)
C8	0.0515 (10)	0.0551 (10)	0.0507 (9)	−0.0137 (8)	0.0115 (8)	−0.0167 (8)
C9	0.0299 (8)	0.0678 (12)	0.0603 (10)	0.0029 (7)	0.0095 (7)	−0.0120 (9)
C10	0.0586 (11)	0.0703 (12)	0.0397 (8)	0.0009 (9)	0.0288 (8)	−0.0049 (8)

### Geometric parameters (Å, °)

**Table:**

Ni1—N1	2.0619 (11)	C2—H2A	0.9300
Ni1—N1^i^	2.0619 (11)	C3—C4	1.4387 (18)
Ni1—N4^i^	2.0753 (15)	C5—C7	1.523 (2)
Ni1—N4	2.0753 (15)	C5—C8	1.530 (2)
Ni1—O1	2.0831 (10)	C5—C6	1.563 (2)
Ni1—O1^i^	2.0832 (10)	C6—C10	1.5243 (19)
S1—C1	1.7040 (16)	C6—C9	1.527 (2)
S1—C3	1.7204 (13)	C7—H7A	0.9600
O1—N2	1.2880 (14)	C7—H7B	0.9600
O2—N3	1.2756 (17)	C7—H7C	0.9600
N1—C3	1.3222 (16)	C8—H8A	0.9600
N1—C2	1.3669 (17)	C8—H8B	0.9600
N2—C4	1.3398 (18)	C8—H8C	0.9600
N2—C5	1.5055 (16)	C9—H9A	0.9600
N3—C4	1.3520 (16)	C9—H9B	0.9600
N3—C6	1.5047 (19)	C9—H9C	0.9600
N4—N5	1.1745 (19)	C10—H10A	0.9600
N5—N6	1.1547 (19)	C10—H10B	0.9600
C1—C2	1.351 (2)	C10—H10C	0.9600
C1—H1	0.9300		
			
N1—Ni1—N1^i^	180.00 (5)	N2—C4—C3	127.35 (11)
N1—Ni1—N4^i^	91.22 (5)	N3—C4—C3	123.63 (12)
N1^i^—Ni1—N4^i^	88.78 (5)	N2—C5—C7	109.04 (11)
N1—Ni1—N4	88.78 (5)	N2—C5—C8	105.60 (12)
N1^i^—Ni1—N4	91.22 (5)	C7—C5—C8	109.74 (15)
N4^i^—Ni1—N4	180.0	N2—C5—C6	101.18 (10)
N1—Ni1—O1	88.32 (4)	C7—C5—C6	115.82 (12)
N1^i^—Ni1—O1	91.68 (4)	C8—C5—C6	114.50 (12)
N4^i^—Ni1—O1	90.39 (7)	N3—C6—C10	109.03 (13)
N4—Ni1—O1	89.61 (7)	N3—C6—C9	106.66 (13)
N1—Ni1—O1^i^	91.68 (4)	C10—C6—C9	109.73 (13)
N1^i^—Ni1—O1^i^	88.32 (4)	N3—C6—C5	101.08 (10)
N4^i^—Ni1—O1^i^	89.61 (7)	C10—C6—C5	115.53 (13)
N4—Ni1—O1^i^	90.38 (7)	C9—C6—C5	114.01 (13)
O1—Ni1—O1^i^	180.0	C5—C7—H7A	109.5
C1—S1—C3	89.29 (7)	C5—C7—H7B	109.5
N2—O1—Ni1	124.18 (8)	H7A—C7—H7B	109.5
C3—N1—C2	110.91 (11)	C5—C7—H7C	109.5
C3—N1—Ni1	125.52 (9)	H7A—C7—H7C	109.5
C2—N1—Ni1	123.11 (9)	H7B—C7—H7C	109.5
O1—N2—C4	127.15 (11)	C5—C8—H8A	109.5
O1—N2—C5	119.91 (11)	C5—C8—H8B	109.5
C4—N2—C5	112.82 (10)	H8A—C8—H8B	109.5
O2—N3—C4	123.76 (12)	C5—C8—H8C	109.5
O2—N3—C6	123.12 (11)	H8A—C8—H8C	109.5
C4—N3—C6	112.60 (11)	H8B—C8—H8C	109.5
N5—N4—Ni1	129.20 (13)	C6—C9—H9A	109.5
N6—N5—N4	178.30 (19)	C6—C9—H9B	109.5
C2—C1—S1	110.99 (11)	H9A—C9—H9B	109.5
C2—C1—H1	124.5	C6—C9—H9C	109.5
S1—C1—H1	124.5	H9A—C9—H9C	109.5
C1—C2—N1	114.84 (12)	H9B—C9—H9C	109.5
C1—C2—H2A	122.6	C6—C10—H10A	109.5
N1—C2—H2A	122.6	C6—C10—H10B	109.5
N1—C3—C4	122.97 (11)	H10A—C10—H10B	109.5
N1—C3—S1	113.94 (10)	C6—C10—H10C	109.5
C4—C3—S1	122.99 (9)	H10A—C10—H10C	109.5
N2—C4—N3	108.88 (11)	H10B—C10—H10C	109.5
			
N1—Ni1—O1—N2	−21.29 (12)	O1—N2—C4—N3	177.25 (14)
N1^i^—Ni1—O1—N2	158.71 (12)	C5—N2—C4—N3	−6.89 (15)
N4^i^—Ni1—O1—N2	69.92 (13)	O1—N2—C4—C3	1.4 (2)
N4—Ni1—O1—N2	−110.08 (13)	C5—N2—C4—C3	177.22 (13)
O1^i^—Ni1—O1—N2	−114 (16)	O2—N3—C4—N2	−178.14 (14)
N1^i^—Ni1—N1—C3	177 (16)	C6—N3—C4—N2	−6.19 (16)
N4^i^—Ni1—N1—C3	−70.05 (12)	O2—N3—C4—C3	−2.1 (2)
N4—Ni1—N1—C3	109.95 (12)	C6—N3—C4—C3	169.88 (12)
O1—Ni1—N1—C3	20.30 (11)	N1—C3—C4—N2	−2.8 (2)
O1^i^—Ni1—N1—C3	−159.70 (11)	S1—C3—C4—N2	173.34 (11)
N1^i^—Ni1—N1—C2	−11 (16)	N1—C3—C4—N3	−178.16 (13)
N4^i^—Ni1—N1—C2	101.39 (12)	S1—C3—C4—N3	−1.98 (19)
N4—Ni1—N1—C2	−78.61 (12)	O1—N2—C5—C7	−45.32 (18)
O1—Ni1—N1—C2	−168.26 (11)	C4—N2—C5—C7	138.49 (14)
O1^i^—Ni1—N1—C2	11.74 (11)	O1—N2—C5—C8	72.54 (16)
Ni1—O1—N2—C4	15.2 (2)	C4—N2—C5—C8	−103.65 (14)
Ni1—O1—N2—C5	−160.42 (9)	O1—N2—C5—C6	−167.85 (12)
N1—Ni1—N4—N5	148.00 (19)	C4—N2—C5—C6	15.96 (14)
N1^i^—Ni1—N4—N5	−32.00 (19)	O2—N3—C6—C10	−50.34 (19)
N4^i^—Ni1—N4—N5	21 (16)	C4—N3—C6—C10	137.65 (14)
O1—Ni1—N4—N5	−123.68 (18)	O2—N3—C6—C9	68.08 (18)
O1^i^—Ni1—N4—N5	56.32 (18)	C4—N3—C6—C9	−103.93 (14)
Ni1—N4—N5—N6	−172 (100)	O2—N3—C6—C5	−172.49 (14)
C3—S1—C1—C2	−0.63 (12)	C4—N3—C6—C5	15.50 (15)
S1—C1—C2—N1	−0.18 (17)	N2—C5—C6—N3	−17.26 (12)
C3—N1—C2—C1	1.19 (18)	C7—C5—C6—N3	−134.97 (13)
Ni1—N1—C2—C1	−171.36 (10)	C8—C5—C6—N3	95.77 (14)
C2—N1—C3—C4	174.82 (12)	N2—C5—C6—C10	−134.76 (13)
Ni1—N1—C3—C4	−12.85 (18)	C7—C5—C6—C10	107.53 (16)
C2—N1—C3—S1	−1.67 (15)	C8—C5—C6—C10	−21.73 (19)
Ni1—N1—C3—S1	170.66 (6)	N2—C5—C6—C9	96.76 (13)
C1—S1—C3—N1	1.34 (11)	C7—C5—C6—C9	−20.95 (18)
C1—S1—C3—C4	−175.14 (12)	C8—C5—C6—C9	−150.21 (14)

Symmetry codes: (i) −*x*+1, −*y*+2, −*z*+1.
